# Effect of Tryptophan Hydroxylase-2 rs7305115 SNP on suicide attempts risk in major depression

**DOI:** 10.1186/1744-9081-6-49

**Published:** 2010-08-25

**Authors:** Yuqi Zhang, Changsong Zhang, Guozhen Yuan, Jianjun Yao, Zaohuo Cheng, Chaojun Liu, Qinghai Liu, Gairong Wan, Guizhi Shi, Yiren Cheng, Yang Ling, Ke Li

**Affiliations:** 1 Wuxi Psychiatric Hospital, Nanjing Medical University, Wuxi, China; 2 Ankang Hospital, Jining, China; 3 Clinical Oncology Laboratory, Changzhou Tumor Hospital, Medical College of Suzhou University, Changzhou, China; 4 Department of Molecular Epidemiology, Shantou University Medical College, Shantou, China

## Abstract

**Background:**

Suicide and major depressive disorders (MDD) are strongly associated, and genetic factors are responsible for at least part of the variability in suicide risk. We investigated whether variation at the tryptophan hydroxylase-2 (TPH2) gene rs7305115 SNP may predispose to suicide attempts in MDD.

**Methods:**

We genotyped TPH2 gene rs7305115 SNP in 215 MDD patients with suicide and matched MDD patients without suicide. Differences in behavioral and personality traits according to genotypic variation were investigated by logistic regression analysis.

**Results:**

There were no significant differences between MDD patients with suicide and controls in genotypic (AG and GG) frequencies for rs7305115 SNP, but the distribution of AA genotype differed significantly (14.4% vs. 29.3%, *p *< 0.001). The G-allele frequency was significantly higher in cases than control group (58.1% vs.45.6%, *p *< 0.001), but the A-allele carrier indicated a decreased trend in MDD with suicide behaviors than control group (41.9% vs.54.4%, *p *< 0.001). The multivariate logistic regression analysis indicated that TPH2 rs7305115 AA (OR 0.33, 95% CI 0.22-0.99), family history of suicide (OR 2.98, 95% CI 1.17-5.04), negative life events half year ago (OR 6.64, 95% CI 2.48-11.04) and hopelessness (OR 7.68, 95% CI 5.79-13.74) were significantly associated with the suicide behaviors in MDD patients.

**Conclusions:**

The study suggested that hopelessness, negative life events and family history of suicide were risk factors of attempted suicide in MDD while the TPH2 rs7305115A remained a significant protective predictor of suicide attempts.

## Background

Suicide is an important public health problem and ranks among the top 10 causes of death for individuals of all ages and major depressive disorders (MDD) appeared to confer greater risk for suicide [1,2]. Suicidal behavior is commonly considered to result from an interaction of genetic, neurobiological, and psychosocial factors. Genetic risk factors are estimated to account for approximately 30% to 40% of the variance in suicidal behavior, however the precise mechanism of the genetic contribution are unknown [3].

Dysregulation of brain serotonin contributes to many psychiatric disorders. Furthermore, abnormal serotonergic function has frequently been reported in individuals who commit or attempt suicide and is one of the most replicated findings in modern biological psychiatry [4]. For example, low levels of serotonin (5-hydroxytryptamine, 5-HT) have also been observed in suicide victims and 5-HT could play a role in the predisposition to suicide [5]. Tryptophan hydroxylase (TPH), the rate limiting enzyme in the biosynthesis of 5-HT neurotransmission, is a major candidate for genetic association studies in many psychiatric disorders, including suicide [6,7]. Two genes coding TPH (TPH1 and TPH2) have been differentiated. The human TPH2 gene is located on chromosome 12q15, comprises 11 exons, and covers a region of about 93.5 kilobases (gene accession number: NM_173353). TPH2, rather than TPH1, is preferentially expressed in the brain [8]. TPH2 is neuron-specific and expressed predominantly in serotonergic neurons of the raphe nuclei and in the peripheral myenteric neurons in the gut [9,10]. The genetic polymorphisms affecting TPH2 gene expression might result in the alteration of physiological processes related to 5-HT. 5-HT is involved in the dysfunction of numerous psychiatric disorders and behavioral traits, such as MDD, suicide, or depression.

To date, nearly five hundred SNPs have been identified in human TPH2, most of them located in non-coding regions of the gene. However, a few functional polymorphisms have been reported. A functional (C1473G) SNP in mouse TPH2 that results in the substitution of Pro447 with Arg447 and leads to decreased serotonin levels in PC12 cells provides direct evidence for TPH2 controls brain serotonin synthesis [11]. Zill et al provides evidence for an involvement of genetic variants of the TPH2 gene in the pathogenesis of MDD and might be a hint on the repeatedly discussed duality of the serotonergic system [12]. The human TPH2 promoter polymorphism rs11178997 impacts on TPH2 expression, which might have implications for the development and function of the serotonergic system in the brain [13]. The TPH2 gene and its 5' upstream region variants (SNPs: rs4448731 and rs4641527) may be involved in the predisposition to suicide in MDD [14]. The core promoter of human TPH2 was localized to the region between -107 and +7, and the segment of +8 to +53 within the 5'-UTR was found to exert a potent inhibitory effect on gene expression at both transcriptional and post-transcriptional levels [15]. The TPH2 C2755A polymorphism may represent a population-specific risk factor for peripartum major depression and anxiety disorder, perhaps by interacting with hormones in Chinese [16]. These results may open up new research strategies for the analysis of the observed disturbances in the serotonergic system in patients suffering from several other psychiatric disorders. Understanding the mechanisms of TPH2 gene polymorphism in suicide attempters may shed light on the neurobiology of the vulnerability to suicidal behavior and reveal potential targets of preventive and therapeutic actions.

In the current study, we investigated another TPH2 polymorphism named rs7305115 at approximately 1077 bp from the 7 exon. To our knowledge, its functionality has not been studied so far. Therefore, the purpose of the present study was to detect possible association between rs7305115 polymorphisms of TPH2 gene and suicide behavior in MDD patients. We performed association and linkage disequilibrium studies on 215 MDD who committed suicide and 215 control MDD patients without suicide behavior. It could be an attempt to examine the association between different components-genetic, environmental, neurobiological, and behavioral of a complex, multivariate and heterogeneous phenomenon.

## Materials and methods

### Patients and controls

The sample investigated consisted of 430 unrelated MDD patients who were recruited from Mar 2004 to May 2008 in the Han nationality of Shandong Province, China. All patients (184 males, 246 females) hospitalized in psychiatric clinics in Shandong, China. Diagnosis of MDD should be confirmed by the Mini International Neuropsychiatric Interview and by a minimum score of Hamilton Depression Rating Scale (HDRS) [17,18]. All cases met DSM-IV diagnostic criteria for MDD and were severe enough to require follow-up in a specialized psychiatric outpatient clinic [19,20]. Axis I and Axis II psychopathologies were determined using the Structured Clinical Interview for DSM-IV Axis I Disorders (SCID-I) and the Structured Clinical Interview for DSM-IV Personality Disorders (SCID-II), respectively. The SCID was administered to all patient subjects by experienced psychiatrists. By means of structured questionnaires, information on specific demographic and clinical variables, such as family history of suicide and history of physical or sexual abuse, was also obtained.

Two hundred and fifteen MDD patients (defined as MDD+suicide group, 92 males, and 123 females) consecutively admitted to our psychiatric departments after a suicide were included in this study. The suicide patient was defined as intentional self-harm to end one's life but not die as a result of his/her action(s). Individuals were scored as positive for a history of suicide attempt only when medical records documented a severe attempt, a relative or partner con- firmed the history, or there was physical evidence confirming the history. We classified suicide methods as violent or nonviolent according to the classification used in previous study [21]. Drug overdose, carbon monoxide poisoning, and drowning were considered nonviolent. All other methods were classified as violent.

Two hundred and fifteen MDD control patients (defined as MDD-suicide group, 92 males, and 123 females) without suicide attempt, which were closely matched with individual patients in the suicidal group in terms of age and gender, were selected for this study. They met all the DSM-IV criteria for major depressive disorder and had HDRS scored of over 18. They had no previous history of suicide attempt or family history of suicide. The other exclusion criteria included evidence of alcohol or drug dependency, significant organic brain disease, and clinically significant somatic disease.

All cases and control subjects were the Han population and came from the same geographical area in eastern China. The protocol of the study was in accordance with the ethics standards of the committee on Human Experimentation of Nanjing Medical University.

### Genotyping

Venous blood was drawn and immediately frozen in aliquots at -70°C or below until analyzed. For genotyping, genomic DNA was extracted from EDTA blood samples by using a commercial DNA extract kit, Wizard Genomic DNA purification kit (Promega, Madison, WI, USA). TPH2 rs7305115 SNP genotyping was performed by DNA sequencing. First, DNA was amplified by polymerase chain reaction (PCR). PCR were carried out in 25 ul volumes containing 20 ng genomic DNA, 0.4 mM primers, 50 mM KCL, 10 mM Tris/HCl (pH 8.3), 0.025% Tween 20, 0.025 mg/ml BSA, 1.5 mM magnesium chloride, 0.4 mM dNTP and 1 U Taq polymerase with the following oligonucleotide primers: forward 5'-ACCTGAGCCCACGAGACTTT-3'; reverse 5'-TCGAGCCAGAGCTGGAATAT-3'. After an initial denaturation step for 5 min at 94°C, 35 cycles of denaturing at 94°C for 30 s, annealing at 55°C for 30 s and extension of 72°C for 30 s were performed, followed by a final extension step of 72°C for 5 min. After it had been amplified, the 312 bp PCR products was subjected to direct sequencing on an ABI 3700 automated DNA sequencer using a Dye Terminator Cycle sequencing kit (Applied Biosystems, Foster City, CA).

### Statistical analysis

The differences in genotype distributions between patients and controls and the Hardy-Weinberg equilibrium (HWE) of each marker were analyzed using a chi-square test. Differences in age and age of onset between three groups were calculated with t-test. Allelic and genotypic frequency distributions were compared between groups by chi-square tests for independence. The univariate and multivariate logistic regression analyses were performed to evaluate the unique contribution of identified risk factors in the prediction of suicide behaviors. Possible interactions between genetic variants and other risk factors were also investigated using logistic regression analyses. Odds ratio (OR) values and 95% confidence intervals (CI) were calculated. The results were considered nominally significant at the level of *p *< 0.05. The SPSS package (version 13.0 for Windows; SPSS, Chicago, Illinois) was used for statistical analyses.

## Results

### Clinical characteristics of patients

The demographic and clinical characteristics of the patients are presented in Table 1. The study population consisted of 215 MDD+suicide (mean age ± SD, 33.4 ± 11.9 years) and 215 matched MDD-suicide (mean age ± SD, 33.6 ± 10.8 years). For MDD+suicide group, one hundred and seventy-one patients (79.5%) suicided by drug overdose, hanging, drowning and 44 patients (20.5%) suicided by violent methods such as several deep cuts. No statistically significant differences were observed between the MDD+suicide and MDD-suicide groups for age, age at onset, course of disease or HDRS scores.

**Table 1:** Demographic Characteristics of the MDD patients with suicide and MDD patients without suicide

	MDD+suicide	MDD-suicide	*p *value
Sex (males/females)	92/123	92/123	
Age (years, mean ± SD)	15 ~ 75 (33.4 ± 11.9)	15 ~ 77 (33.6 ± 10.8)	0.721
Age of onset (years, mean ± SD)	14 ~ 75 (28.4 ± 10.3)	15 ~ 76 (29.4 ± 10.7)	0.565
Course of disease (years, mean ± SD)	2 weeks ~ 25 years (3.43 ± 4.52)	2 weeks ~ 29 years (3.39 ± 4.54)	0.935
HDRS scores ^a^	51.19 ± 10.61	49.87 ± 10.49	0.079

^a ^HDRS, Hamilton depression rating scale for depression

### Genotypes and allele frequencies in the TPH2 rs7305115 polymorphisms

**Figure 1:**
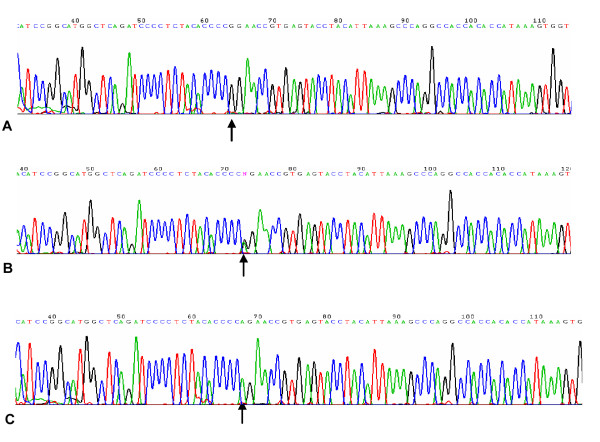
**Schematic representation of TPH2 rs7305115 SNP**. Arrows represent homozygous genotypes GG (A), AA (B) and heterozygous genotype AG (C) of TPH2 rs7305115 SNP investigated in this study.

A total of 430 unrelated, major depression patients with and without suicide behaviors were genotyped for the TPH2 rs7305115 polymorphisms. The distributions of both TPH2 polymorphisms in MDD+suicide and MDD-suicide were in agreement with the Hardy-Weinberg equilibrium (HWE) applying the HWSIM computer program http://krunch.med.yale.edu/hwsim. The distributions of genotypes for TPH2 rs7305115 polymorphisms did not deviate from HWE in MDD with or without suicide groups (Table 2). Homozygous genotypes were identified by the presence of GG and AA, but the AG for heterozygous genotype (Figure 1). In the total sample, there were no significant differences between MDD+suicide and MDD-suicide in genotypic (AG and GG) frequencies for TPH2 rs7305115 polymorphisms. But the distribution of AA genotype differed significantly between MDD-suicide and MDD+suicide groups (29.3% vs.14.4%, *p *< 0.001). Meanwhile, the distribution of AA genotype was significantly lower than AG+GG group in MDD+suicide groups (14.4% vs. 85.6%, *p *< 0.001). The allele distributions of both A and G was significant differences between MDD+suicide and MDD-suicide in our sample. The G-allele frequency in MDD+suicide was higher than MDD-suicide (58.1% vs.45.6%, *p *< 0.001), but the A-allele carrier indicated a decreased trend in MDD with suicide behaviors by compared with MDD-suicide group (41.9% vs.54.4%, *p *< 0.001). We suggested that the presence of the A-allele was a significant predictor for suicide behaviors because of the low frequency of the AA genotype in MDD+suicide group.

**Table 2:** Distribution of genotypes and allele frequencies of TPH2 rs7305115 polymorphism

		MDD+suicide (n = 215)	MDD-suicide (n = 215)	*P value ^a^*	OR (95%CI)
Genotype (N, %)				**< 0.001**	**--**
	AA	31 (14.4%)	63 (29.3%)		
	AG	118 (54.9%)	108 (50.2%)		
	GG	66 (30.7%)	44 (20.5%)		
Genotype (N, %)				**< 0.001**	0.406
	AA	31 (14.4%)	63 (29.3%)		(0.251-0.657)
	AG+GG	184 (85.6%)	152 (70.7%)		
Allele (N, %)				**< 0.001**	0.603
	A	180 (41.9%)	234 (54.4%)		(0.461-0.790)
	G	250 (58.1%)	196 (45.6%)		

^a ^MDD+suicide vs. MDD-suicide, Pearson chi-square test.

### Genotypes of TPH2 rs7305115 polymorphism and HDRS scores in MDD+suicide group

We determined the genotypes of TPH2 rs7305115 polymorphism and HDRS scores in MDD+suicide group (Table 3). The TPH2 rs7305115 SNP was not associated with HDRS scores in sum for MDD+suicide (*p *= 0.092). But, we found that HDRS scores of cognitive impairment and hopelessness were associated with TPH2 rs7305115 genotypes among MDD+suicide (*p *= 0.035 and *p *= 0.032). The patients with AA genotype indicated lower HDRS scores for cognitive impairment (mean ± SD, 7.90 ± 3.97) and hopelessness (mean ± SD, 9.60 ± 2.12) factors than AG and GG groups in MDD with suicide behaviors. This suggested that the TPH2 rs7305115 AA could be a significant protective predictor for suicide behaviors.

**Table 3:** Genotypes of TPH2 rs7305115 polymorphism and HDRS scores in MDD+suicide group (scores, mean ± SD)

	Genotype			*P *value
		
	AA	AG	GG	
Anxiety/Somatization	l2.83 ± 4.03	l2.11 ± 3.72	l2.01 ± 3.83	0.113
Loss of weight	1.81 ± 0.54	1.78 ± 0.54	1.55 ± 0.48	0.832
Cognitive impairment	7.90 ± 3.97	10.05 ± 4.23	10.93 ± 3.82	**0.035**
Diurnal change	1.76 ± 1.27	1.67 ± 1.26	1.45 ± 0.97	0.223
Slow movement	10.23 ± 2.41	9.15 ± 2.33	9.38 ± 2.75	0.468
Sleep disorders	5.61 ± 0.66	5.24 ± 0.71	5.60 ± 0.85	0.176
Hopelessness	9.60 ± 2.12	11.57 ± 1.80	11.29 ± 2.10	**0.032**
HDRS scores in sum	49.80 ± l0.57	51.49 ± l0.71	52.28 ± l0.83	0.092

### Predictors for the suicide behaviors in MDD patients

To investigate the predictive power of AA genotype of TPH2 rs7305115 polymorphisms, a univariate logistic regression analysis was performed to examine the predictive effect of each factor on the risk for behaviors in MDD patients (Table 4). The 36 potential risk factors for MDD+suicide group were compared with those of MDD-suicide group. Compared with the AG and GG groups, the AA group (OR = 0.50, 95% CI = 0.31-0.82, *p *< 0.001) were at lower risk for suicide attempts. The TPH2 polymorphism emerged as a significant variable that could reliably predict clinical suicide behaviors.

**Table 4:** Results from univariate logistic regression analysis i.e., OR, 95% CI, Wald statistic, and probability values for predictor variables of MDD suicide behaviors

Variables	*OR*	95% *CI*	*Wald*	*P *value
Education under middle school	2.39	1.62-3.52	19.705	**0.000**
Married	0.20	0.12-0.32	46.600	**0.000**
Age of onset (= ≥ 35 years)	0.79	0.54-1.17	1.407	0.236
Short course of diseases (= ≤ 1 year)	0.83	0.57-1.21	0.943	0.332
Employed	0.67	0.45-1.00	3.809	0.051
Family history of Mental illness	0.94	0.64-1.39	0.086	0.769
Onset abruptly or subacute	1.13	0.76-1.68	0.366	0.545
Predisposing factors	1.15	0.78-1.72	0.500	0.479
Unhappy childhood	1.12	0.70-1.80	0.233	0.650
Family history of suicide	2.58	1.47-4.54	11.328	**0.001**
Physical disease	1.15	0.69-1.93	0.279	0.597
Monthly income (= < $40)	2.02	1.37-2.99	7.892	**0.005**
Smoking	1.64	1.02-2.65	4.164	**0.041**
Drinking	1.64	1.07-2.53	5.178	**0.023**
Introversion	1.90	1.25-2.87	9.155	**0.002**
Economic pressure	2.58	1.64-4.05	17.434	**0.000**
Unhappy a month ago	0.96	0.65-1.42	0.040	0.841
Unhappy a year ago	1.14	0.76-1.72	0.397	0.529
Unhappy after a year	0.87	0.60-1.31	0.362	0.547
Friend suicide two weeks ago	1.11	0.46-2.66	0.050	0.823
Friend suicide a year ago	1.22	0.60-2.49	0.295	0.587
Negative life events half year ago	5.74	3.28-10.04	43.365	**0.000**
Family emotional support needed	0.43	0.26-0.72	10.587	**0.001**
Family emotional support gained	1.06	0.72-1.55	0.086	0.769
Friends' emotional support needed	0.38	0.24-0.60	16.025	**0.000**
Friends' emotional support gained	1.06	0.72-1.55	0.084	0.772
Depression (HDRS = ≥ 35) ^a^	1.58	1.07-2.34	5.227	**0.020**
Hopelessness (HDRS = ≥ 6) ^a^	7.68	4.99-11.83	93.755	**0.000**
Anxiety/Somatization(HDRS = > 7) ^a^	0.84	0.56-1.27	0.706	0.401
Loss of weight in a week(= 0.5 kg)	0.93	0.64-1.36	0.149	0.699
Cognitive impairment(HDRS = > 12) ^a^	1.17	0.77-1.78	0.553	0.457
Diurnal change (HDRS = ≥ 1) ^a^	1.12	0.76-1.66	0.350	0.554
Slow movement (HDRS = ≥ 8) ^a^	1.12	1.77-4.64	0.340	0.560
Sleep disorders (HDRS = ≥ 2) ^a^	3.28	2.20-4.88	35.128	**0.000**
Anxiety (HAMA = ≥ 21) ^b^	4.12	2.54-6.68	35.622	**0.000**
TPH2 rs7305115 AA	0.50	0.31-0.82	13.941	**0.000**

^a ^HDRS, Hamilton depression rating scale for depression;

^b ^HAMA, Hamilton anxiety scale for anxiety

Significant risk factors were entered into a forward selection multivariate logistic regression analysis. The involvement of sixteen factors in MDD+suicide was analyzed using multiple logistic regression analysis. Using this model, the degree of involvement of each factor for suicide patients could be estimated (Table 5). On multivariate analysis, the final model indicated that TPH2 rs7305115 AA (OR 0.33, 95% CI 0.22-0.99, P < 0.001), family history of suicide (OR 2.98, 95% CI 1.17-5.04, P < 0.001), negative life events half year ago (OR 6.64, 95% CI 2.48-11.04, P < 0.001) and hopelessness (OR 7.68, 95% CI 5.79-13.74, P < 0.001) were significantly associated with the suicide behaviors in MDD patients. The TPH2 rs7305115A remained a significant protective predictor of suicide behaviors.

**Table 5:** Results from multivariate logistic regression analysis for predictor variables of MDD+suicide group

Variables	*OR*	95% *CI*	*Wald*	*P *value
TPH2 rs7305115 AA	0.33	0.22-0.99	12.861	**0.000**
Family history of suicide	2.98	1.17-5.04	10.228	**0.001**
Negative life events half year ago	6.64	2.48-11.04	41.145	**0.000**
Hopelessness (HDRS > = 6) ^a^	7.68	5.79-13.74	90.235	**0.000**

^a ^HDRS, Hamilton depression rating scale for depression

Further, we examined the interactions between TPH2 genotypes and the potential three factors on the risk for MDD+suicide group (Table 6). The results indicated that family history of suicide, negative life events half year ago and hopelessness were significantly associated with TPH2 genotypes in MDD+suicide patients (*p *= 0.006, *p *= 0.005, and *p *= 0.002, respectively).

**Table 6:** Association analysis of TPH2 genotypes and predictor variables in MDD+suicide group

	Genotype (N)			*P value ^a^*
	AA (n = 31)	AG (n = 118)	GG (n = 66)	
Family history of suicide				**0.006**
Yes	5	45	33	
No	26	73	33	
Negative life events half year ago				**0.005**
Yes	10	51	42	
No	21	67	24	
Hopelessness				**0.002**
Yes	17	86	58	
No	14	32	8	

^a ^Pearson chi-square test.

## Discussion

Suicide receives increasing attention worldwide, with many countries developing national strategies for prevention. Rates of suicide vary greatly between countries, with the greatest burdens in developing countries [22]. Most people who die by suicide have psychiatric disorders, notably mood, substance-related, anxiety, psychotic, and personality disorders, with comorbidity being common [23]. Risk for suicide may have genetic contributions, however, specific genes or relevant DNA sequence variations have not yet been identified [24]. Therefore, it is a major research challenge to clarify the relative heritability of the risk for suicide, in particular, separately from the heritability of disorders or traits that are strongly associated with suicidal risk [25].

The rate-limiting enzyme of serotonin biosynthesis, TPH2, is one of the most promising candidate genes for psychiatric disorders [26]. In the present study, we investigated TPH2 rs7305115 variants in a Han sample of suicide attempters, and compared them to non-suicidal depressed patients. We also investigated the association of polymorphisms with the life environment factors among suicide patients. The issue of association of TPH2 SNPs with suicide-related behavior is a complex and controversial one. Several studies have indicated possible associations between various TPH2 polymorphisms and major depression, suicidal behavior [12,27]. In contrast, other studies failed to find associations between TPH2 polymorphisms and suicidality [28,29]. Nevertheless, we found an association of suicidal behavior with the TPH2 rs7305115 SNP in the present study. Our data shows that MDD+suicide patients have low frequencies of the rs7305115 A-allele and AA genotypes (OR = 0.50).

Studies indicated that a history of suicide attempt, a psychiatric condition such as ongoing major depression, alcohol or other substance use disorder, hopelessness, separation or loss, anger, and suicidal ideation have been implicated as predictors of suicidal behaviors [30,31]. The MDD patients with greater levels of hopelessness also were prone to the multiple suicide attempts [32]. Our findings indicated that cognitive impairment and hopelessness were associated with TPH2 rs7305115 SNP among MDD+suicide patients.

The multivariate analysis indicated that four factors (TPH2, family history of suicide, negative life events half year ago and hopelessness) were significantly associated with the suicide behaviors in MDD patients. The TPH2 rs7305115 AA remained a significant protective predictor of suicide behaviors (OR = 0.33). The results suggested that A→G mutation carriers of TPH2 rs7305115 SNP could be a higher risk of suicide attempts than AA homozygous genotype carriers in MDD patients. To identify the functional locus, it will be important to closely evaluate the significance of sequence variants in coding regions that could alter gene expression at the DNA or RNA levels.

## Limitations

A number of limitations may be relevant to these studies. First, we only paid attention on a SNP maker of the TPH2 gene in the current study. More TPH2 marker will be examined for MDD patients with suicide behaviors in the future study. Further, we had no the detail mechanistic explanation regarding the role of serotonin functioning in depression for TPH2 SNP. Further study is needed to elucidate its mechanism. Understanding why the vulnerability to suicide differs among MDD patients could assist in improved screening of high risk patients and treatment.

## Conclusions

In case-control association studies, our findings suggested that the rs7305115 SNP in the TPH2 gene could be a marker for the genetic susceptibility to suicide-related traits in Chinese. The TPH SNP might influence suicide-related traits in behaviorally extreme populations and included an independent high risk clinical sample, suicide attempters. We suggested that TPH2 gene could regulate serotonergic system and contribute to the vulnerability to suicidal behavior.

## Abbreviations

DSM: The Diagnostic and Statistical Manual of Mental Disorders; HDRS: Hamilton Depression Rating Scale; SCID-I: Structured Clinical Interview for DSM-IV Axis I Disorders; SCID-II: Structured Clinical Interview for DSM-IV Personality Disorders; 5-HT: 5-hydroxytryptamine; TPH2: the tryptophan hydroxylase-2; MDD: major depressive disorders; HWE: Hardy-Weinberg equilibrium.

## Competing interests

The authors declare that they have no competing interests.

## Authors' contributions

JY, ZC, and CL carried out the molecular genetic studies, participated in the sequence alignment and drafted the manuscript. QL, GW, GS, YC, YL, and KL recruited the Subjects. CZ and GY participated in the design of the study and performed the statistical analysis. YZ conceived of the study, and participated in its design and coordination and helped to draft the manuscript. All authors read and approved the final manuscript.
